# An exploratory clinical study of β-glucan combined with camrelizumab and SOX chemotherapy as first-line treatment for advanced gastric adenocarcinoma

**DOI:** 10.3389/fimmu.2024.1448485

**Published:** 2024-08-26

**Authors:** Yunqian Chu, Xuan He, Ya Xue, Hua Jiang, Chan Zhu, Chunjian Qi, Xing Zhang, Dongsheng Chen, Hanjue Dai, Qingying Xian, Wenyu Zhu

**Affiliations:** ^1^ Department of Oncology, The Affiliated Changzhou No.2 People’s Hospital of Nanjing Medical University, Changzhou, China; ^2^ West China Hospital, Sichuan University, Chengdu, China; ^3^ Jiangsu Simcere Diagnostics Co., Ltd., Nanjing Simcere Medical Laboratory Science Co., Ltd., The State Key Laboratory of Neurology and Oncology Drug Development, Nanjing, China; ^4^ Medical Research Center, The Affiliated Changzhou No.2 People’s Hospital of Nanjing Medical University, Changzhou, China

**Keywords:** β-glucan, camrelizumab, chemotherapy, first-line, gastric cancer

## Abstract

**Background:**

β-glucan has been reported to be a potential natural immune modulator for tumor growth inhibition. We aimed to evaluate the efficacy and safety of β-glucan plus immunotherapy and chemotherapy in the first-line treatment of advanced gastric adenocarcinoma.

**Methods:**

This is a phase IB, prospective, single-arm, investigator-initiated trail. Advanced gastric adenocarcinoma patients received β-glucan, camrelizumab, oxaliplatin, oral S-1 every 3 weeks. The curative effect was evaluated every 2 cycles. The primary endpoints were objective response rate (ORR) and safety, with secondary endpoints were median progression-free survival (mPFS) and median overall survival (mOS). The exploratory endpoint explored biomarkers of response to treatment efficacy.

**Results:**

A total of 30 patients had been enrolled, including 20 (66.7%) males and all patients with an ECOG PS score of ≥1. The ORR was 60%, the mPFS was 10.4 months (95% confidence interval [CI], 9.52-11.27), the mOS was 14.0 months (95% CI, 11.09-16.91). A total of 19 patients (63.3%) had TRAEs, with 9 patients (30%) with grade ≥ 3. The most common TRAEs were nausea (53.3%). After 2 cycles of treatment, the levels of IL-2, IFN-γ and CD4+ T cells significantly increased (*P* < 0.05). Furthermore, biomarker analysis indicated that patient with better response and longer OS exhibited lower GZMA expression at baseline serum.

**Conclusions:**

This preliminary study demonstrates that β-glucan plus camrelizumab and SOX chemotherapy offers favorable efficacy and a manageable safety profile in patients with advanced gastric adenocarcinoma, and further studies are needed to verify its efficacy and safety.

**Clinical Trial Registration:**

Chinese Clinical Trials Registry, identifier ChiCTR2100044088

## 1 Introduction

Gastric cancer (GC), including gastroesophageal junction cancer (GEJC), stand as one of the five leading causes of cancer-related deaths worldwide (1). Due to its insidious hidden onset and rapid progression, most patients are diagnosed in the advanced stages, leading to a 5-year survival rate of less than 10% (2). Although the survival rate of patients with advanced GC has improved in recent years, the prognosis remains bleak. Fluorouracil combined with cisplatin or oxaliplatin is the standard first-line chemotherapy for patients with advanced GC (3), but the mOS is still less than 1 year. For the past few years, immune checkpoint inhibitors (ICIs) have shown significant anti-tumor activity in the treatment of various malignant tumors (4, 5), though their efficacy in GC first line treatment remains inconclusive (6–9). For instance, the CheckMate 649 study combined a standardized chemotherapy regimen with immunotherapy (nivolumab), resulting in extended mOS, reduced risk of death, and increased ORR regardless of the level of PD-L1 expression (10, 11). Conversely, the KEYNOTE-062 study found that adding immunotherapy (pembrolizumab) to first-line chemotherapy did not significantly improve PFS or OS compared to chemotherapy alone (8). Therefore, there is an urgent need for more effective therapeutic approaches.

β-glucan, a macromolecular polysaccharide, is a major molecular pattern that stimulates innate and adaptive immune responses (12).As a natural immunomodulator, β-glucan has garnered attention in tumor immunotherapy studies (13–16). Its granular form reduces the inhibitory ability of myeloid-derived suppressor cells through the dectin-1 signaling pathway, activates of dendritic cells (DCs), down-regulates the immunosuppressive effect of regulatory T cells (Tregs), and promotes the proliferation of effector T cells, thereby impeding tumor growth and reducing metastatic ability (17, 18). β-glucan can also inhibit tumor growth by activating the src-Syk-PI3K pathway via iC3b through CR3-dependent cell-mediated cytotoxicity (19). Professor Chavakis’ team confirmed that adoptive transfer of neutrophils from β-glucan-trained mice to naive recipients suppressed tumor growth in the latter in a ROS-dependent manner (20). A recent study found that coupling anti-PD-L1 antibody with β-glucan can induce an earlier immune response, infiltration of DCs, and activation of pre-existing T cells in the tumor microenvironment in mice compared to anti-PD-L1 antibody (21).Additionally, our previous research has indicated that combining β-glucan with ICIs and chemotherapy improves median PFS among patients with advanced cancer exhibiting drug resistance and may even reverse drug resistance (22).

As far as we know, no research has explored β-glucan plus immunotherapy and chemotherapy as a first-line treatment for advanced GC. Hence, we sought to explore whether adding β-glucan to first-line standard chemotherapy plus immunotherapy could produce synergistic effects in patients with advanced GC. We aimed to assess the clinical efficacy and safety of this therapy regimen. Further, we conduct an exploratory cytokines, T-cell subsets and proteomics analysis with the aim of identifying promising biomarkers of treatment response. Here, we show the promising efficacy and manageable safety profile of β-glucan in combination with concurrent immunotherapy and chemotherapy for the first-line treatment of advanced G/GEJ adenocarcinoma.

## 2 Materials and methods

### 2.1 Study design

This phase IB, single-arm, investigator-initiated exploratory study was conducted at the Affiliated Changzhou No.2 People’s Hospital of Nanjing Medical University, Jiangsu, China. Experimental subjects were not randomized into groups and experimenters and patients were not blinded because our study was a pilot exploratory study.

This study received approval from the Institutional Review Board at study center and adhered to the principles of Good Clinical Practice and the Declaration of Helsinki, with monitoring by an academic steering committee. Informed written consent was obtained from each subject or independent witness prior to clinical trial enrollment. This study has been registered with the Chinese Clinical Trials Registry (ChiCTR2100044088).

### 2.2 Patient selection

Patients with advanced gastric or gastroesophageal junction adenocarcinoma were eligible for enrollment between April 2021 and October 2022. The key inclusion criteria were as follows: aged 18 to 85 years; confirmed diagnosis of advanced gastric or gastroesophageal junction adenocarcinoma confirmed by histology or cytology; HER2 negative status; ECOG score ≤2; no previous treatment with chemotherapy; and assessment of ≥ 1 measurable lesion according to Response Evaluation Criteria in Solid Tumors (RECISTv1.1) with sufficient organ function. The main exclusion criteria were as follows: treatment with radiotherapy within 14 days after enrollment; presence of active autoimmune diseases requiring systemic treatment in the past 2 years (excluding hormone replacement therapy); presence of pneumonia or history of noninfectious pneumonia requiring steroids; and presence of active infection requiring systemic treatment.

### 2.3 Treatment

Eligible patients received the following treatment regimen (1): SOX chemotherapy: intravenous oxaliplatin (day 1; 130 mg/m2; infusion time, 3-5 hours) + oral tegafur (twice daily on days 1-14; body surface area < 1.25 m2: 40 mg; body surface area 1.25-1.5 m2: 50 mg; body surface area > 1.5 m2: 60 mg); (2) ICI treatment: intravenous camrelizumab (day 2; fixed-dose 200 mg; infusion time, 45 min); and (3) oral whole glucan particle (WGP) β-glucan (500 mg; twice daily on days 1-14). All drugs were administered every 3 weeks as a cycle until the occurrence of disease progression, death, or intolerable toxicity occurred.

### 2.4 Study endpoints and assessments

The primary endpoints were ORR and safety, while secondary endpoints included mPFS and mOS. Imaging examinations were performed every 2 cycles, tumor response was assessed according to RECISTv1.1. CR was defned as complete disappearance of the tumor with no new lesions. PR was defned as≥30% decrease in the longest diameter of the target lesions and no progression in new lesions. SD was defned as<30% decrease or <20% increase in the target lesions and no progression in new lesions. PD was defned as≥20% increase in the longest diameter of the target lesions or the appearance of new lesions. ORR was defined as the sum of CR and PR, disease control rate (DCR) was defined as the sum of CR, PR, and SD. PFS was defined from the time from the first day of use of the regimen initiation to disease progression or death, and OS was defined as the time from the date of from enrollment to the last follow-up confirmation of death. Treatment response was assessed every 6 weeks (± 7 days), and treatment-related adverse events (TRAEs) were monitored and graded using the National Cancer Institute Common Terminology Criteria for Adverse Events (Version 4.0).

### 2.5 Detection of PD-L1 expression

For patients providing fresh tumor tissue or frozen specimens, the expression of PD-L1 was assessed via immunohistochemistry (PD-L1 IHC 22C3 antibody). The comprehensive positive score (CPS) was used to interpret the expression level of PD-L1. To obtain this score, the number of PD-L1–positive cells was divided by the total number of tumor cells, and this result was multiplied by 100. When this final number was ≥1, PD-L1 expression was defined as positive.

### 2.6 Biomarker analysis

Blood samples were collected after enrollment and at the first evaluation of treatment efficacy, with 8ml of blood collected each time. From these samples, cytokines were detected using the RAISECARE kit (Reskel Biotechnology Co., Ltd.). This panel allows simultaneous quantification of the detection of 12 key targets: IL-1β, IL-2, IL-4, IL-5, IL-6, IL-8, IL-10, IL-12p70, IL-17, IFN-α, IFN-γ, and TNF-α. The number of CD4+ T cells (CD3+, CD4+, CD8–) and CD8+ T cells (CD3+, CD4–, CD8+) in the peripheral blood was assessed using CD3FITC, CD8aPerCT, and CD4APC antibodies (Beijing Saitaike Biotechnology Co., Ltd.) and flow cytometry.

According to the guidelines provided by the manufacturer, plasma samples were analyzed using Olink Target Immuno-Oncology panel (Olink Proteomics, Sweden), which includes detection of 92 proteins by the Proximity Expansion Assay (PEA). The assay was performed by Jiangsu Simcere Diagnostics Co. Ltd. The procedure included sample addition, hybridization incubation, extension and amplification, and data analysis. The resulting cycling thresholds (Ct) were quality controlled and normalized using three negative controls for calculating the limit of detection (LOD) and three interplate controls (IPC) containing group 92 antibodies. Log2 was used to convert Olink data that had been extracted in Normalized Protein Expression (NPX) units. The expression levels of metabolism-related target proteins in patient serum were examined using Olink-targeted proteomics technology, and the obtained NPX was analyzed between groups.

### 2.7 Statistical analysis

The measurement data were tested for normality. If the data followed a normal distribution, a paired t test was used for comparisons. If the data did not follow a normal distribution, a Wilcoxon rank-sum test was used for comparisons. When *P* < 0.05, the difference was considered statistically significant. The Kaplan-Meier survival method was used to analyze PFS and OS. IBM SPSS software (V22.0) and GraphPad Prism (V9.0) were used for analysis and plotting. We did not check for sample sizes using a power analysis because our study does not report statistics on between groups or within group variables.

## 3 Results

### 3.1 Patient characteristics

A total of 36 patients with GC or GEJC were initially included in the study. Among them, three patients were ultimately excluded because of protocol issues, and another three patients were lost to follow-up after one or two treatment cycles. Consequently, the final study population consisted of 30 patients, with a median age of 67.5 years. Of these participants, 20 (66.7%) were male. None of the patients with an ECOG performance status score of 0, all had scores of 1 (77.3%) or 2 (26.1%). Most patients (80%) had GC, and nearly half (46.7%) had previously undergone radical surgery. In addition, the majority (93.3%) had metastasis disease, with liver metastasis accounting for 46.7%. PD-L1 expression was positive in 56.7% of patients (**Table 1**).

**Table 1:** Baseline characteristics of patients (n = 30).

Characteristic	Value
Median age, y (53-81)	67.5 (53-81)
Sex, *n* (%)	
Male	20 (66.7)
Female	10 (33.3)
ECOG (screening phase), *n* (%)	
1	22 (73.3)
2	8 (26.7)
Primary location, *n* (%)	
Gastric	24 (80.0)
Gastroesophageal junction	6 (20.0)
History of surgery, *n* (%)	
Yes	14 (46.7)
No	16 (53.3)
Disease state, *n* (%)	
Metastasis	28 (93.3)
Local progression/recurrence	2 (6.7)
Liver metastasis	14 (46.7)
PD-L1 CPS, *n* (%)	
<1	5 (16.7)
≥1	17 (56.7)
Unknown	8 (26.7)

CPS, comprehensive positive score; ECOG, Eastern Cooperative Oncology Group; PD-L1, programmed death ligand 1.

### 3.2 Treatment delivery

All 30 patients received at least 2 cycles of the prescribed treatment regimen, with 20 patients (66.7%) completed at least 6 cycles. At the time of data collection, the median follow-up time was 9.4 months (range, 3.7-18.6 months). The most common cause of treatment discontinuation was disease progression, noted in two patients (8.3%).

### 3.3 Treatment efficacy

**Figure 1:**
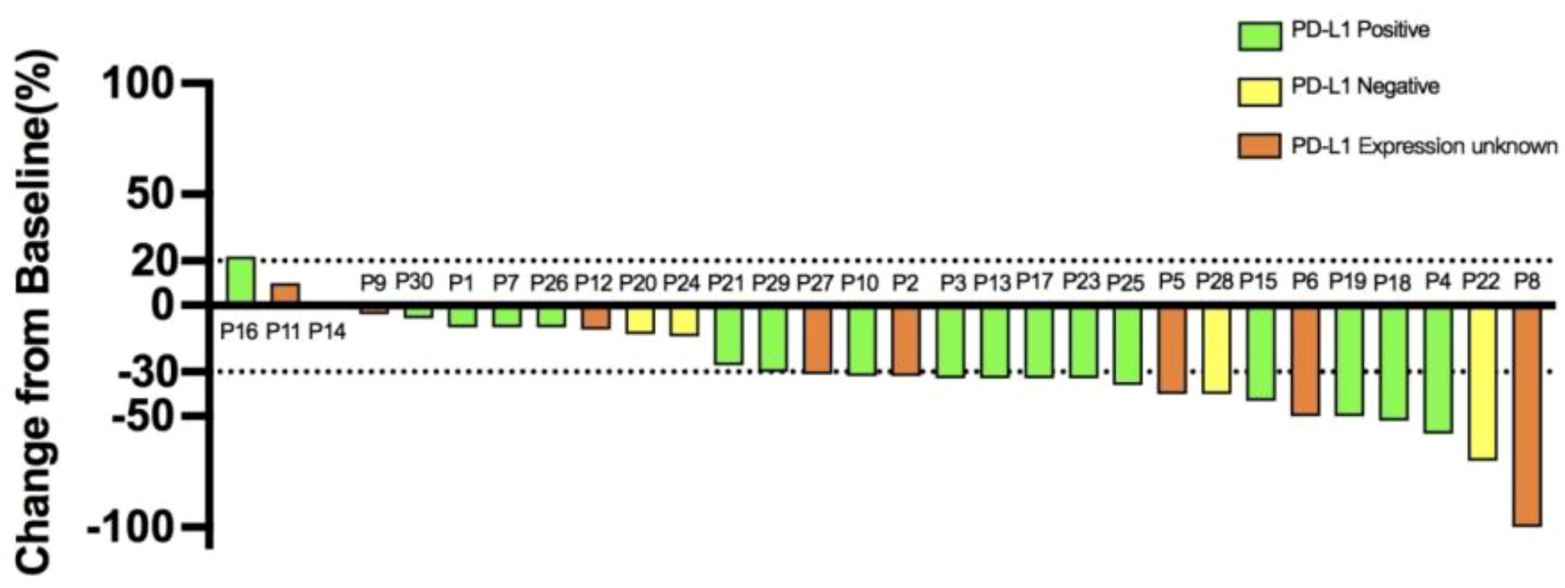
Waterfall plot of the best overall response to treatment in the patients with gastric cancer. Each bar indicates the percentage change from baseline in the sum of the diameters of the target lesion.

**Figure 2:**
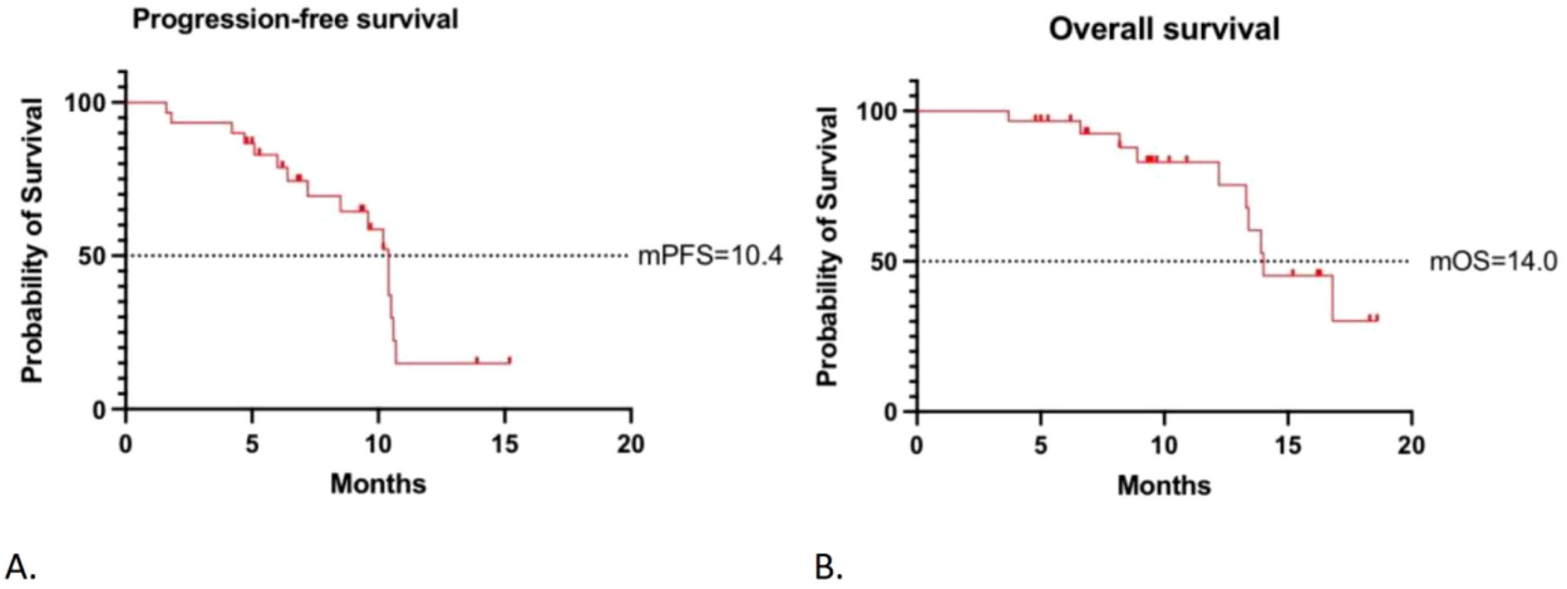
Survival outcome of all patients. Kaplan-Meier survival curves for **(A)** progression-free survival (PFS) and **(B)** overall survival (OS).

Upon data collection, one patient (3.3%) demonstrated CR, 17 patients (56.7%) demonstrated PR, 10 patients (33.3%) demonstrated SD, and two patients (6.7%) demonstrated PD. The ORR was 18 (60.0%; 95% confidence interval [CI], 41.4%-78.6%), and the DCR was 28 (93.3%; 95% CI, 77.9%-99.2%). In 28 of the study patients (93.3%), the total target lesion diameter at the time of best overall response to treatment was smaller than the lesion diameter at baseline (**Figure 1**). The mPFS was 10.4 months (95% CI, 9.52-11.27), and the mOS was 14.0 months (95% CI, 11.09-16.91; **Figure 2**).

Two patients discontinued treatment because they met the criteria for surgical resection. One patient had GC with peritoneal lymph node metastasis, after 4 cycles of treatment, the gastric mass and peritoneal lymph node metastasis were both considerably reduced, with the response classified as PR. Subsequently, the patient underwent surgical resection, resulting in a disease stage of ypT1N0M0 (stage IA). The second patient had GC with peritoneal lymph node metastasis, after 4 cycles of treatment, the response to therapy was classified as SD, the primary lesion had reached the standard of surgical resection and radical gastrectomy was performed. The postoperative disease was staged as ypT1N1M0 (IIA). Both patients received maintenance therapy and adjuvant chemotherapy post-surgery, with continued monitoring of their OS.

### 3.4 Safety profile

During treatment period, 19 (63.3%) patients had ≥ 1 treatment-related adverse event, with 9 patients (30.0%) demonstrating AEs of grade 3 or higher (**Table 2**). The most common AEs were nausea (53.3%) and decreased appetite (50.0%). The most common AEs of grade 3 or higher were neutropenia (13.3%) and anemia (13.3%). Grade 3 or higher AEs were alleviated with appropriate clinical treatment. Ten patients (33.3%) had AEs specifically related to the use of camrelizumab, namely hemangioma; 1 of these patients underwent hemangioma resection. No patients terminated treatment because of AEs, and no adverse event–related deaths occurred. No AEs related to oral β-glucan were observed.

**Table 2:** Treatment-related adverse events during treatment in patients (n= 30).

Adverse event	Any grade	Grade ≥3
Hematologic, *n* (%)		
Decrease in neutrophil count	12 (40.0)	4 (13.3)
Decrease in platelet count	12 (40.0)	2 (6.7)
Anemia	6 (20.0)	4 (13.3)
Decrease in white blood cell count	5 (16.7)	1 (3.3)
Nonhematologic, *n* (%)		
Nausea	16 (53.3)	0
Decreased appetite	15 (50.0)	1 (3.3)
Vomiting	5 (16.7)	1 (3.3)
Increase in alanine aminotransferase level	5 (16.7)	0
Increase in aspartate aminotransferase level	5 (16.7)	0
Fatigue	5 (16.7)	0
Diarrhea	3 (10.0)	0
Other, *n* (%)		
Angioma	10 (33.3)	1 (3.3)

### 3.5 Detection of cytokines and T-cell subsets

**Figure 3:**
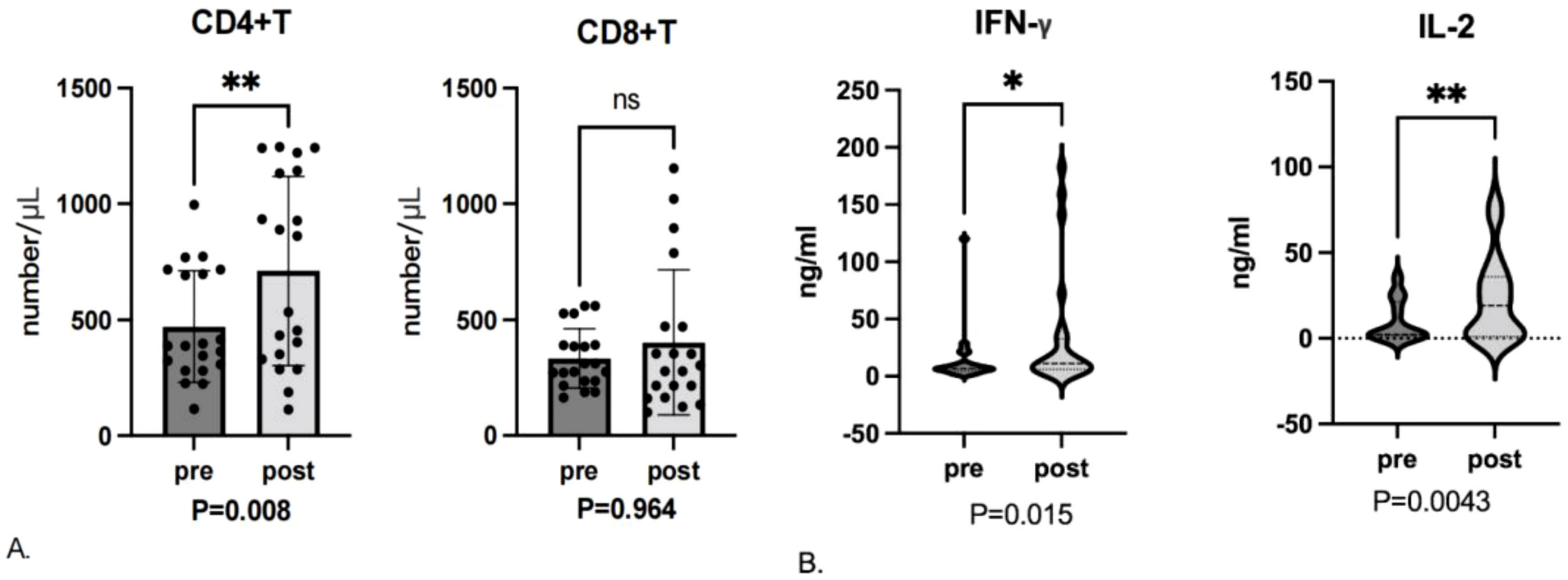
**(A)** T-cell subset levels assessed before enrollment (pre) and after the first efficacy evaluation (post). ns, not significant. **(B)** Cytokine levels assessed before enrollment and after the first efficacy evaluation. * represent *p* ≤ 0.05, ** represent *p* ≤ 0.01.

Of the 30 study patients, 20 had blood samples obtained before enrollment and during the first efficacy evaluation (6 patients refused). Blood samples from 20 patients were analyzed for T-cell subsets (the remaining patients had substandard samples). After 2 cycles of therapy, the number of CD4+ T cells had increased significantly from baseline (*P* = 0.008; **Figure 3A**). Although the number of CD8+T cells trended higher after treatment, this difference was not significant (*P* = 0.964).

Analysis of changes in cytokine levels from the 24 patients with blood samples available demonstrated significant increases in IL-2 (*P* = 0.0043) and IFN-γ (*P* = 0.015) levels after treatment (**Figure 3B**). Although trends were seen for changes in the remaining cytokine levels, these differences were not significant.

### 3.6 Serum GZMA expression at baseline was associated with patient response and PFS

Baseline and post-treatment blood samples from a total of 13 (17 patients had missing samples) patients were analyzed by Olink Target 96 Inflammation panel, which measures levels of 92 marker proteins in key immune and inflammatory pathways. Of the 13 patients, 1 patient had a CR and 2 patients achieved a PR were considered responders. Nine patients had SD and one patient obtained PD were considered non-responders. Patients with better response to treatment had a longer trend in PFS compared to non-responders (**Supplementary Figure 1A**, p = 0.101), but there was no statistical trend in OS (**Supplementary Figure 1B**, p = 0.49).

The comparison of protein levels before and after treatment showed a dynamic change in systemic immune proteomics. As shown in **Supplementary Figures 1C, D**, ten proteins were differentially expressed post-treatment versus pre-treatment (p<0.05), all differentially expressed proteins were downregulated after treatment (CXCL1, CXCL5, IL7, IL8, MCP-1, MCP-3, MMP12, PDGF subnitB, TNFRSF12A) were significantly lower in serum after treatment compared to prior treatment (*p*<0.05), except for the PDCD1 protein, it indicated that β-glucan combined with immunochemical therapy induced complex systemic immune response.

**Figure 4:**
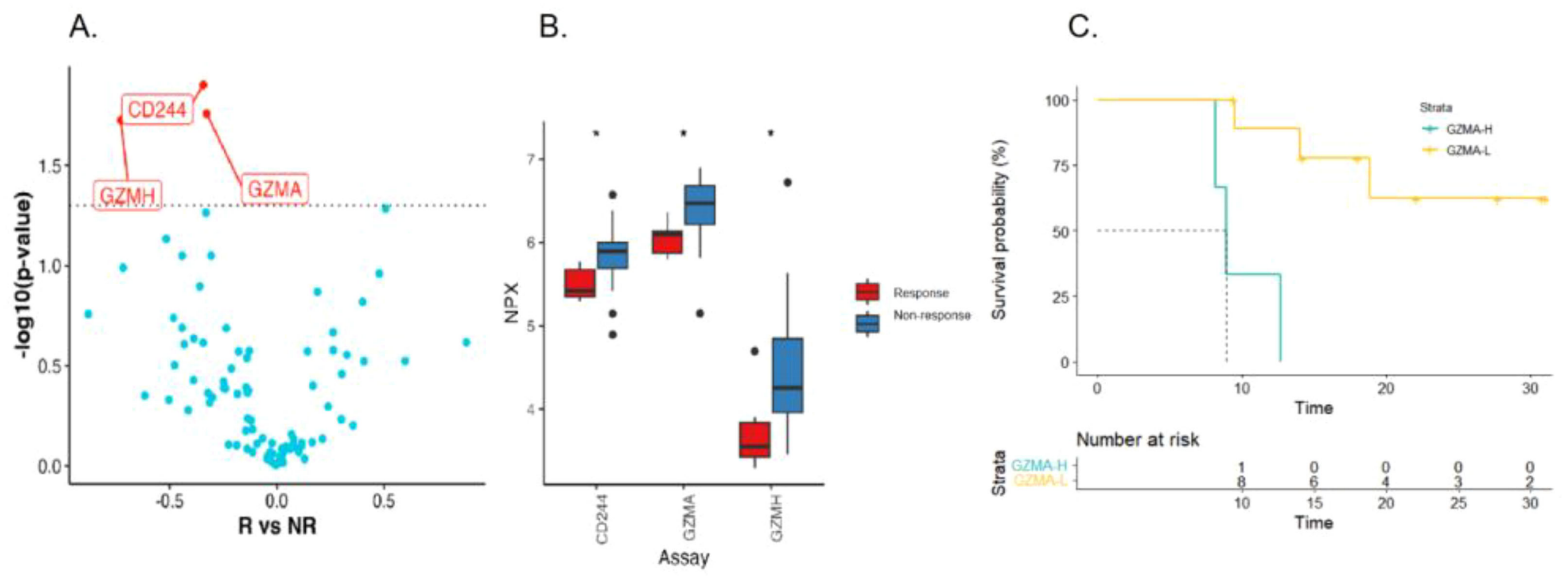
Serum immunoproteomics is associated with therapeutic response and prognosis. Volcanic **(A)** and box plot **(B)** of changes in baseline serum protein levels in responding and non-responding patients (R: NR= 3:11). **(C)** Kaplan-Meier curves of OS for patients with high and low baseline GZMA level.

We further compared baseline serum protein levels in patients with different treatment responses in an attempt to find markers of therapeutic efficacy. Three proteins were differentially expressed in response and non-response group (*p*<0.05, **Figures 4A, B**). All differentially expressed proteins were significantly lower in response group (GZMA, GZMH and CD244) than that of non-response group. In order to identify possible predictors of survival, and the first quartile value of the cohort (n=13) is used as the critical value. As shown in **Figure 4C** of survival analysis using Kaplan-Meier method, patients with high GZMA expression had a tendency to have poorer OS (log rank test *p* = 0.001). Combined with clinical pathological factors, we performed univariate and multivariable COX analysis and found that high GZMA expression was associated with worse OS (*p* = 0.013, HR = 18.363, 95%CI 1.853-181.962, **Supplementary Table 1**). In summary, low expression of GZMA is a positive biomarker of response and prognosis in patients with advanced gastric cancer receiving first-line β-glucan combined immunochemical therapy.

## 4 Discussion

In this study, we assessed the efficacy and safety of a novel immunomodulator (β-glucan) plus immunotherapy and chemotherapy for patient with advanced GC. Our finding reveal that this treatment regimen yielded an ORR of 60%, an mPFS of 10.4 months, and an mOS of 14.0 months, with an acceptable safety profile. These preliminary results suggest the potential utility of this treatment regimen as a therapeutic option for advanced GC.

Patients demonstrated an ORR of 60% in our study. While this falls slightly below the 66.7% reported in ATTRACTION-4 study of nivolumab plus SOX chemotherapy (23), but 47.6% of the patients in ATTRACTION-4 study had an ECOG score of 0, and the remaining patients had an ECOG score of 1. In contrast, our study enrolled patients with poorer physical status, none of whom had an ECOG score of 0, and a notable proportion (26.7%) had an ECOG score of 2. Patients in the current our study demonstrated an mPFS of 10.4 (95% CI, 9.52-11.27) months, surpassing the mPFS of 7.7 reported in the CheckMate-649 study (24). Another phase III clinical study (the Orient-16 study) demonstrated that patients with a PD-L1 CPS >5 had an mPFS of 7.7 months, compared to only 7.1 months for the entire study population, with an ORR of 58.2% (25). These findings suggest potential advantages of the β-glucan plus immunotherapy plus chemotherapy over these other therapies for advanced GC. OUR previous research has shown that β-glucan combined with ICIs can delay tumor invasion in a mouse model of lung cancer, improve mPFS among patients with advanced cancer in whom drug resistance has developed, and even reverse drug resistance to some extent (22). This may partially explain the increased PFS and ORR we observed in this study. The mOS in the current study was 14.0 months compared to a mOS of 15.2 months in the Orient-16 study. The higher ECOG scores in patient population may have affected this result. Several studies have shown that the presence of liver metastasis alters the efficacy of immunotherapy, with these patients demonstrating a significantly worse prognosis than those without liver metastasis (26–29). Nearly half of patients (46.7%) in this study presented with liver metastasis at baseline, which may be another reason affecting mOS. Lee et al. (30) showed that the liver has a role in promoting long-term tumor antigen-specific immunosuppression, which may be the mechanism for the poorer mOS in patients with liver metastases in this study.

The number of AEs reported in this study was consistent with the number typically seen with first-line therapy in this patient population (31). The types of immune-related AEs in this study were consistent with those reported in the KEYNOTE-062 study (32), and no patients discontinued treatment or died due to AEs. The occurrence of special adverse reactions (hemangiomas) was mainly related to the use of camrelizumab. Grade 1 and 2 AEs responded well to appropriate clinical treatment, suggesting that the combination treatment used in this study is an appropriate option for patients with GC.

In this study, we analyzed cytokine and T-cell subset levels in patients. The number of CD4+ T cells also increased significantly after 2 cycles of treatment, but the change in CD8+ T cell levels was not significant. Berner et al. (33) suggested that oral β-glucan can significantly increase cytotoxic T lymphocytes (CTL) in spleen. A recent study also found that WGP β-glucan can induce up-regulation of CD4+ T cells, down-regulate Treg cells, and improve the immunosuppressive tumor microenvironment (34). IL-2 and IFN-γ levels were found to change significantly with treatment. Previous research has shown that β-glucan can be used to stimulate innate and adaptive immune responses (35, 36).β-glucan can bind to TLT/MR/Dectin-1/CR3 receptors; activate downstream MAPK, NF-κB, and other signaling pathways; activate and promote the proliferation of immune cells such as natural killer cells, DCs, and T cells; and increase the release of IFN-γ, IL-2, TNF-α, granzyme B, and perforin (37). Our results support these previous findings. However, other research has shown that IL-2 has a bidirectional immune regulatory function, that the signal transmission mediated by IL-2/IL-2R (IL-2 receptor) is important for the differentiation and development of Treg cells, and that IL-2 therefore has a negative immune regulatory effect (38). These findings suggest that changes in IL-2 levels and the potential effects of these changes require further analysis.

To further elucidate the predictive indicators for therapy efficacy and prognosis through systemic changes in the immune environment, we conducted serum proteomics testing. We have included clinical factors in COX univariate and multivariate regression model to analyze the effect of GZMA protein expression levels on survival. We found that high GZMA expression (top 25%) was associated with worse OS in Cox multivariate regression (forward: conditional). Therefore, high serum GZMA expression at baseline revealed to be an independent factor for first-line immunotherapy combined with chemotherapy for gastric cancer. However, the exact mechanism has not been clarified. A study found that GZMA expression increased in colon tissue of mice with CRC progression. In a mouse model, deficiency of extracellular GZMA both attenuate gut inflammation and prevent CRC development through cell transformation and epithelial-to-mesenchymal transition. Targeted inhibition of GZMA can improve the prognosis of CRC patients (39). Low expression of GZMA may still be a positive biomarker of response and prognosis in patients for first-line combination therapy with β-glucan in gastric cancer.

This study had several limitations. The results we obtained regarding changes in Serum samples cannot be directly linked to the use of β-glucan, as ICIs and chemotherapy can also alter the tumor immune environment. In addition, this was a single-arm exploratory study with a limited sample size, and no control group for comparative analysis. Comparisons were made for immune indicators obtained from blood samples after enrollment and at the time of the first efficacy evaluation may not capture all instances of immune activation, requiring dynamic analysis using multinode blood sample assays. PD-L1 expression was deficient in some patients in our study. Therefore, when we attempted to analyze the survival of the remaining patients based on negative or positive PD-L1 expression, we were unable to do so using log-rank due to the small number of patients and the fact that approximately half of the patients had not yet reached the PFS node. We intend to continue to follow up until all study patients have reached the PFS node to complete this analysis.

## 5 Conclusion

These preliminary results suggest that in patients with advanced GC, even those with poor ECOG performance, β-glucan combined with camrelizumab and SOX chemotherapy offers considerable clinical benefits and a manageable safety profile. Additional studies including control groups and larger patient populations are needed to further explore these initial findings.

## Data Availability

The original contributions presented in the study are included in the article/**Supplementary Material**. Further inquiries can be directed to the corresponding author.

## References

[1] Sung H Ferlay J Siegel RL Laversanne M Soerjomataram I Jemal A. Global cancer statistics 2020: GLOBOCAN estimates of incidence and mortality worldwide for 36 cancers in 185 countries. CA: Cancer J Clin. (2021) 71:209–49. doi: 10.3322/caac.21660 33538338

[2] Li P Huang CM Zheng CH Russo A Kasbekar P Brennan MF. Comparison of gastric cancer survival after R0 resection in the US and China. J Surg Oncol. (2018) 118:975–82. doi: 10.1002/jso.25220 PMC6319936 30332517

[3] Ajani JA D'Amico TA Bentrem DJ Chao J Cooke D Corvera C. Gastric cancer, version 2.2022, NCCN clinical practice guidelines in oncology. J Natl Compr Cancer Network: JNCCN. (2022) 20:167–92. doi: 10.6004/jnccn.2022.0008 35130500

[4] Wang Z Chen T Lin W Zheng W Chen J Huang F. Functional tumor specific CD8 + T cells in spleen express a high level of PD-1. Int immunopharmacology. (2020) 80:106242. doi: 10.1016/j.intimp.2020.106242 32014811

[5] Inno A Roviello G Ghidini A Luciani A Catalano M Gori S. Rechallenge of immune checkpoint inhibitors: A systematic review and meta-analysis. Crit Rev oncology/hematology. (2021) 165:103434. doi: 10.1016/j.critrevonc.2021.103434 34343657

[6] Shitara K Özgüroğlu M Bang YJ Di Bartolomeo M Mandalà M Ryu MH. Pembrolizumab versus paclitaxel for previously treated, advanced gastric or gastro-oesophageal junction cancer (KEYNOTE-061): a randomised, open-label, controlled, phase 3 trial. Lancet (London England). (2018) 392:123–33. doi: 10.1016/s0140-6736(18)31257-1 29880231

[7] Shitara K Van Cutsem E Bang YJ Fuchs C Wyrwicz L Lee KW. Efficacy and safety of pembrolizumab or pembrolizumab plus chemotherapy vs chemotherapy alone for patients with first-line, advanced gastric cancer: the KEYNOTE-062 phase 3 randomized clinical trial. JAMA Oncol. (2020) 6:1571–80. doi: 10.1001/jamaoncol.2020.3370 PMC7489405 32880601

[8] Chung HC Kang YK Chen Z Bai Y Wan Ishak WZ Shim BY. Pembrolizumab versus paclitaxel for previously treated advanced gastric or gastroesophageal junction cancer (KEYNOTE-063): A randomized, open-label, phase 3 trial in Asian patients. Cancer. (2022) 128:995–1003. doi: 10.1002/cncr.34019 34878659 PMC9299889

[9] Satoh T Kang YK Chao Y Ryu MH Kato K Cheol Chung H. Exploratory subgroup analysis of patients with prior trastuzumab use in the ATTRACTION-2 trial: a randomized phase III clinical trial investigating the efficacy and safety of nivolumab in patients with advanced gastric/gastroesophageal junction cancer. Gastric cancer: Off J Int Gastric Cancer Assoc Japanese Gastric Cancer Assoc. (2020) 23:143–53. doi: 10.1007/s10120-019-00970-8 PMC6942596 31087200

[10] Janjigian YY Ajani JA Moehler M Shen L Garrido M Gallardo C. First-line nivolumab plus chemotherapy for advanced gastric, gastroesophageal junction, and esophageal adenocarcinoma: 3-year follow-up of the phase III checkMate 649 trial. J Clin oncology: Off J Am Soc Clin Oncol. (2024) 42:2012–20. doi: 10.1200/jco.23.01601 PMC11185916 38382001

[11] Li K Zhang A Li X Zhang H Zhao L. Advances in clinical immunotherapy for gastric cancer. Biochim Biophys Acta Rev cancer. (2021) 1876:188615. doi: 10.1016/j.bbcan.2021.188615 34403771

[12] Lee SH Jang GY Kim MY Hwang IG Kim HY Woo KS. Physicochemical and in *vitro* binding properties of barley β-glucan treated with hydrogen peroxide. Food Chem. (2016) 192:729–35. doi: 10.1016/j.foodchem.2015.07.063 26304404

[13] Qi C Cai Y Gunn L Ding C Li B Kloecker G. Differential pathways regulating innate and adaptive antitumor immune responses by particulate and soluble yeast-derived β-glucans. Blood. (2011) 117:6825–36. doi: 10.1182/blood-2011-02-339812 PMC3128477 21531981

[14] Liu M Tong Z Ding C Luo F Wu S Wu C. Transcription factor c-Maf is a checkpoint that programs macrophages in lung cancer. J Clin Invest. (2020) 130:2081–96. doi: 10.1172/jci131335 PMC7108920 31945018

[15] Tian J Ma J Ma K Guo H Baidoo SE Zhang Y. β-Glucan enhances antitumor immune responses by regulating differentiation and function of monocytic myeloid-derived suppressor cells. Eur J Immunol. (2013) 43:1220–30. doi: 10.1002/eji.201242841 23424024

[16] Ding J Ning Y Bai Y Xu X Sun X Qi C. β-Glucan induces autophagy in dendritic cells and influences T-cell differentiation. Med Microbiol Immunol. (2019) 208:39–48. doi: 10.1007/s00430-018-0556-z 30088084

[17] Tian J Ma J Ma K Ma B Tang X Baidoo SE. Up-regulation of GITRL on dendritic cells by WGP improves anti-tumor immunity in murine Lewis lung carcinoma. PloS One. (2012) 7:e46936. doi: 10.1371/journal.pone.0046936 23077535 PMC3471954

[18] Daley D Mani VR Mohan N Akkad N Ochi A Heindel DW. Dectin 1 activation on macrophages by galectin 9 promotes pancreatic carcinoma and peritumoral immune tolerance. Nat Med. (2017) 23:556–67. doi: 10.1038/nm.4314 PMC5419876 28394331

[19] Albeituni SH Yan J. The effects of β-glucans on dendritic cells and implications for cancer therapy. Anti-cancer Agents medicinal Chem. (2013) 13:689–98. doi: 10.2174/1871520611313050003 23092290

[20] Kalafati L Kourtzelis I Schulte-Schrepping J Li X Hatzioannou A Grinenko T. Innate immune training of granulopoiesis promotes anti-tumor activity. Cell. (2020) 183:771–85.e12. doi: 10.1016/j.cell.2020.09.058 33125892 PMC7599076

[21] Wang Q Jiang H Zhang H Lu W Wang X Xu W. β-Glucan-conjugated anti-PD-L1 antibody enhances antitumor efficacy in preclinical mouse models. Carbohydr polymers. (2024) 324:121564. doi: 10.1016/j.carbpol.2023.121564 37985066

[22] Wang M Bai Y Pei J Li D Pu X Zhu W. β-glucan combined with PD-1/PD-L1 checkpoint blockade for immunotherapy in patients with advanced cancer. Front Pharmacol. (2022) 13:887457. doi: 10.3389/fphar.2022.887457 35548349 PMC9084312

[23] Boku N Ryu MH Kato K Chung HC Minashi K Lee KW. Safety and efficacy of nivolumab in combination with S-1/capecitabine plus oxaliplatin in patients with previously untreated, unresectable, advanced, or recurrent gastric/gastroesophageal junction cancer: interim results of a randomized, phase II trial (ATTRACTION-4). Ann oncology: Off J Eur Soc Med Oncol. (2019) 30:250–8. doi: 10.1093/annonc/mdy540 PMC6386029 30566590

[24] Janjigian YY Shitara K Moehler M Garrido M Salman P Shen L. First-line nivolumab plus chemotherapy versus chemotherapy alone for advanced gastric, gastro-oesophageal junction, and oesophageal adenocarcinoma (CheckMate 649): a randomised, open-label, phase 3 trial. Lancet (London England). (2021) 398:27–40. doi: 10.1016/s0140-6736(21)00797-2 34102137 PMC8436782

[25] Xu J Jiang H Pan Y Gu K Cang S Han L. LBA53 Sintilimab plus chemotherapy (chemo) versus chemo as first-line treatment for advanced gastric or gastroesophageal junction (G/GEJ) adenocarcinoma (ORIENT-16): First results of a randomized, double-blind, phase III study. Ann Oncol. (2021) 32:S1331. doi: 10.1016/j.annonc.2021.08.2133

[26] Tumeh PC Hellmann MD Hamid O Tsai KK Loo KL Gubens MA. Liver metastasis and treatment outcome with anti-PD-1 monoclonal antibody in patients with melanoma and NSCLC. Cancer Immunol Res. (2017) 5:417–24. doi: 10.1158/2326-6066.Cir-16-0325 PMC5749922 28411193

[27] Pires da Silva I Lo S Quek C Gonzalez M Carlino MS Long GV. Site-specific response patterns, pseudoprogression, and acquired resistance in patients with melanoma treated with ipilimumab combined with anti-PD-1 therapy. Cancer. (2020) 126:86–97. doi: 10.1002/cncr.32522 31584722

[28] Schmid S Diem S Li Q Krapf M Flatz L Leschka S. Organ-specific response to nivolumab in patients with non-small cell lung cancer (NSCLC). Cancer immunology immunotherapy: CII. (2018) 67:1825–32. doi: 10.1007/s00262-018-2239-4 PMC11028265 30171269

[29] Topalian SL Hodi FS Brahmer JR Gettinger SN Smith DC McDermott DF. Five-year survival and correlates among patients with advanced melanoma, renal cell carcinoma, or non-small cell lung cancer treated with nivolumab. JAMA Oncol. (2019) 5:1411–20. doi: 10.1001/jamaoncol.2019.2187 PMC6659167 31343665

[30] Lee JC Mehdizadeh S Smith J Young A Mufazalov IA Mowery CT. Regulatory T cell control of systemic immunity and immunotherapy response in liver metastasis. Sci Immunol. (2020) 5:eaba0759. doi: 10.1126/sciimmunol.aba0759 33008914 PMC7755924

[31] Yamada Y Higuchi K Nishikawa K Gotoh M Fuse N Sugimoto N. Phase III study comparing oxaliplatin plus S-1 with cisplatin plus S-1 in chemotherapy-naïve patients with advanced gastric cancer. Ann oncology: Off J Eur Soc Med Oncol. (2015) 26:141–8. doi: 10.1093/annonc/mdu472 25316259

[32] Tabernero J Cutsem EV Bang YJ Fuchs CS Shitara K. Pembrolizumab with or without chemotherapy versus chemotherapy for advanced gastric or gastroesophageal junction (G/GEJ) adenocarcinoma: The phase III KEYNOTE-062 study. J Clin Oncol. (2019) 37:LBA4007–LBA. doi: 10.1200/JCO.2019.37.18_suppl.LBA4007

[33] Berner VK duPre SA Redelman D Hunter KW. Microparticulate β-glucan vaccine conjugates phagocytized by dendritic cells activate both naïve CD4 and CD8 T cells. vitro Cell Immunol. (2015) 298:104–14. doi: 10.1016/j.cellimm.2015.10.007 26549577

[34] Ding C Shrestha R Zhu X Geller AE Wu S Woeste MR. Inducing trained immunity in pro-metastatic macrophages to control tumor metastasis. Nat Immunol. (2023) 24:239–54. doi: 10.1038/s41590-022-01388-8 PMC10636755 36604547

[35] Lipinski T Fitieh A St Pierre J Ostergaard HL Bundle DR Touret N. Enhanced immunogenicity of a tricomponent mannan tetanus toxoid conjugate vaccine targeted to dendritic cells via Dectin-1 by incorporating β-glucan. J Immunol (Baltimore Md: 1950). (2013) 190:4116–28. doi: 10.4049/jimmunol.1202937 23514738

[36] Tang X Huang J Xiong H Zhang K Chen C Wei X. Anti-tumor effects of the polysaccharide isolated from tarphochlamys affinis in H22 tumor-bearing mice. Cell Physiol biochemistry: Int J Exp Cell physiology biochemistry Pharmacol. (2016) 39:1040–50. doi: 10.1159/000447811 27537353

[37] Sahasrabudhe NM Dokter-Fokkens J de Vos P. Particulate β-glucans synergistically activate TLR4 and Dectin-1 in human dendritic cells. Mol Nutr Food Res. (2016) 60:2514–22. doi: 10.1002/mnfr.201600356 27358258

[38] Cheng G Yu A Malek TR. T-cell tolerance and the multi-functional role of IL-2R signaling in T-regulatory cells. Immunol Rev. (2011) 241:63–76. doi: 10.1111/j.1600-065X.2011.01004.x 21488890 PMC3101713

[39] Santiago L Castro M Sanz-Pamplona R Garzón M Ramirez-Labrada A Tapia E. Extracellular granzyme A promotes colorectal cancer development by enhancing gut inflammation. Cell Rep. (2020) 32:107847. doi: 10.1016/j.celrep.2020.107847 32640217

