# Supplementary material for: An exploratory clinical study of β-glucan combined with camrelizumab and SOX chemotherapy as first-line treatment for advanced gastric adenocarcinoma
Source: Front Immunol. 2024 Aug 26;15:1448485. doi: 10.3389/fimmu.2024.1448485 (PMC11381272; doi:10.3389/fimmu.2024.1448485)
Supplement: Supplementary file 1 [file DataSheet1.docx]

Supplementary Material


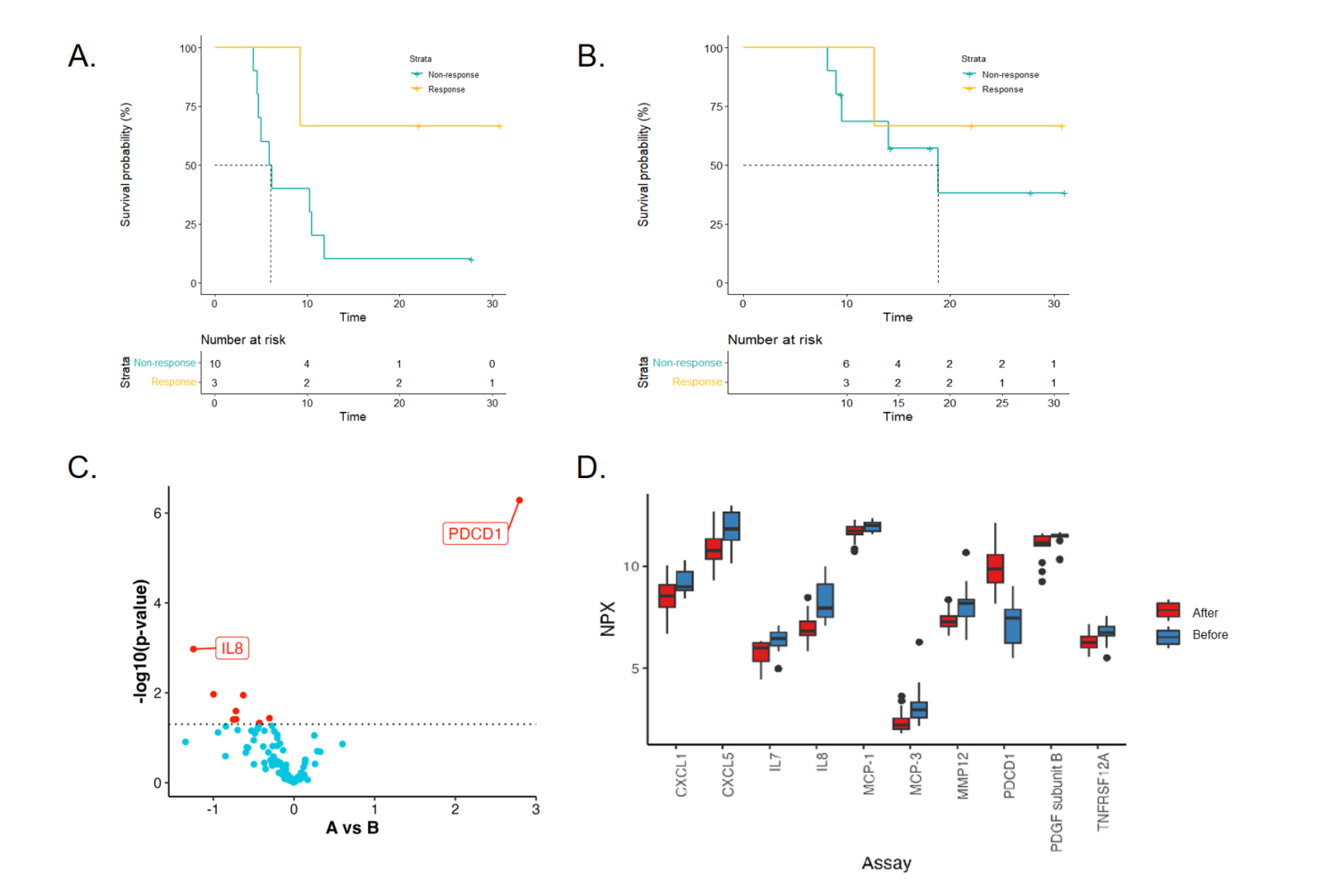
**Supplementary Figure 1.** Kaplan-Meier curves of PFS (A) and OS (B) for response and non-response patients. Volcano (C) and box plot (D) of serum protein levels before and after treatment (n=13 pairs).

**Supplementary Table 1.** The results of univarible and multivarible analysis on GZMA expression with OS.

|  | **Univarible** | | | **Multivariable** | | |
| --- | --- | --- | --- | --- | --- | --- |
| **Characteristic** | **HR^1^** | **95% CI^2^** | **p-value** | **HR^1^** | **95% CI^2^** | **p-value** |
| **Age group (years)** |  |  |  |  |  |  |
| >65 |  |  |  |  |  |  |
| ≤65 | 5.563 | 0.901-34.333 | 0.065 |  |  | 0.622 |
| **Sex group** |  |  |  |  |  |  |
| Female |  |  |  |  |  |  |
| Male | 0.375 | 0.043-3.260 | 0.374 |  |  | 0.057 |
| **ECOG score** |  |  |  |  |  |  |
| 1 |  |  |  |  |  |  |
| 2 | 3.713 | 0.732-18.826 | 0.113 |  |  | 0.472 |
| **Smoking history** |  |  |  |  |  |  |
| Never |  |  |  |  |  |  |
| Always | 33.639 | 0.014-83219.016 | 0.378 |  |  | 0.248 |
| **Drinking history** |  |  |  |  |  |  |
| Never |  |  |  |  |  |  |
| Always | 1.038 | 0.189-5.692 | 0.996 |  |  | 0.31 |
| **Clinical stage** |  |  |  |  |  |  |
| Ⅲ |  |  |  |  |  |  |
| Ⅳ | 1.569 | 0.286-8.611 | 0.604 |  |  | 0.092 |
| **Tumor location** |  |  |  |  |  |  |
| cardia |  |  |  |  |  |  |
| body | 0.78 | 0.140-4.332 | 0.776 |  |  | 0.165 |
| **Number of metastasis** |  |  |  |  |  |  |
| <3 |  |  |  |  |  |  |
| ≥3 | 3.009 | 0.495-18.292 | 0.232 |  |  | 0.766 |
| **GZMA** |  |  |  |  |  |  |
| GZMA-H (top25%) |  |  |  |  |  |  |
| GZMA-L | 0.054 | 0.005-0.540 | 0.013 | 18.363 | 1.853-181.962 | 0.013 |
| ^1^HR = Hazard Ratio; ^2^CI = Confidence Interval. | | | |  |  |  |
